# ALYREF-JunD-SLC7A5 axis promotes pancreatic ductal adenocarcinoma progression through epitranscriptome-metabolism reprogramming and immune evasion

**DOI:** 10.1038/s41420-024-01862-2

**Published:** 2024-02-24

**Authors:** Qingbo Meng, Yuting Xie, Kang Sun, Lihong He, Hongkun Wu, Qi Zhang, Tingbo Liang

**Affiliations:** 1 Department of Hepatobiliary and Pancreatic Surgery, The First Affiliated Hospital, School of Medicine, Zhejiang University, Hangzhou, Zhejiang China; 2 Zhejiang Provincial Key Laboratory of Pancreatic Disease, The First Affiliated Hospital, School of Medicine, Zhejiang University, Hangzhou, Zhejiang China; 3 The Innovation Center for the Study of Pancreatic Diseases of Zhejiang Province, Hangzhou, Zhejiang China; 4 Cancer Center, Zhejiang University, Hangzhou, Zhejiang China; 5 MOE Joint International Research Laboratory of Pancreatic Diseases, Zhejiang University, Hangzhou, Zhejiang China

**Keywords:** Cancer metabolism, Cancer microenvironment, Immunosurveillance

## Abstract

Pancreatic ductal adenocarcinoma (PDAC) is a kind of tumor lacking nutrients due to its poor vascularity and desmoplasia. Recent studies have shown that cancer cells might achieve growth advantage through epitranscriptome reprogramming. However, the role of m^5^C in PDAC was not fully understood. We found that Aly/REF export factor (ALYREF), a reader of m^5^C modification, was overexpressed in PDAC, and associated with bad prognosis. In addition, the ALYREF expression was negatively related to CD8^+^ T cells infiltration in clinical samples. ALYREF knockdown decreased tumor growth in vivo partly dependent of immunity. ALYREF silencing decreased SLC7A5 expression and subsequently inactivated mTORC1 pathway, resulting in decreased tumor proliferation. Mechanically, ALYREF specifically recognized m^5^C sites in JunD mRNA, maintained the stabilization of JunD mRNA and subsequently upregulated transcription of SLC7A5. Since SLC7A5 was a key transporter of large neutral amino acids (LNAAs), overexpression of SLC7A5 on tumor cells depleted amino acid in microenvironment and restricted CD8^+^ T cells function. Moreover, ALYREF-JunD-SLC7A5 axis was overexpressed and negatively related with survival through TMA assays. In conclusion, this research revealed the relationship between m^5^C modification, amino acid transportation and immune microenvironment. ALYREF might be a novel target for PDAC metabolic vulnerability and immune surveillance.

## Introduction

Pancreatic ductal adenocarcinoma (PDAC) is one of the most lethal diseases [1] and only 10–15% of patients are eligible for surgical resection [2]. Compared with other types of cancer, PDAC is relatively chemotherapy-resistant, and survival benefits are very modest. Moreover, targeted therapies and immune checkpoint inhibitors play limited roles in PDAC therapy [3]. Hence, it is imperative to explore new molecular and biological mechanisms of PDAC to identify effective therapeutic strategies and diagnostic biomarkers.

The rapid development of high-throughput sequencing technologies have revealed an emerging mechanism of gene regulation, the epitranscriptome [4]. Epitranscriptomic regulation focuses on post-transcriptional modifications of coding and non-coding RNA. To date, over 170 different types of RNA modifications that affect the function and homeostasis of RNA were identified [5]. Furthermore, increasing evidence demonstrates that epitranscriptome dysregulation plays an important role in tumorigenesis [6]. Recently, several studies suggested that m^5^C modification of mRNA was associated with tumorigenesis and progression. For example, in bladder cancer, the m^5^C modifications of heparin-binding growth factor (HDGF) mRNA are deposited by NSUN2 and are specifically recognized by YBX1 to maintain its stability, thereby driving tumor progression [7]. Nevertheless, the role of m^5^C in PDAC is still elusive and requires further investigation.

Metabolic changes in cancer cells fulfill the biosynthetic demands induced by cell proliferation and survival under nutrient limitations. Metabolic reprogramming is commonly observed in many types of cancers and is considered a significant hallmark of tumors [8]. Besides serving as the substrates of protein, amino acids also participate in energy supply, redox balance and synthesis of nucleotides. To satisfy the demand of exponential growth and proliferation, tumor cells frequently overexpress some enzymes or transporters associated with amino acids. For example, high expression of BCAT1 in nasopharyngeal carcinoma promotes cancer cells invasion [9]. In addition, ATF4 activation mediates apatinib resistance by upregulating ASNS in non-small-cell lung cancer [10]. Adequate nutrition importation is essential not only for tumor cells but also for T cells [11]. Activation, differentiation, and proliferation of T cells are accompanied by increased metabolic requirements. In tumor microenvironment, tumor cells take competitive advantage in amino acids uptake, which diminishes functionality of T cells. Owing to the dependency and addiction on amino acids, targeting amino acid metabolism represents a dual antitumor effect which inhibit the malignant properties of cancer cells as well as tumor-derived immunosuppression. Therefore, identification of specific amino acid regulators in PDAC provides a new insight into therapeutic strategy.

In this study, we demonstrated that upregulation of Aly/REF export factor (ALYREF) in PDAC tissues correlated with poor prognosis and immune escape. In addition, ALYREF promoted PDAC cells proliferation in vitro and in vivo, and restricted the effector function of CD8^+^ T cells. Mechanistically, we found that ALYREF specifically recognized m^5^C sites of JunD mRNA and maintain its stabilization. Then JunD transcriptionally activated SLC7A5 and further enhanced the activity of mTORC1 signaling. Moreover, upregulated SLC7A5 spurred PDAC cells to uptake excessive amino acids which simultaneously disrupt the metabolism of CD8^+^ T cells and restrained their functions. In summary, this research revealed the relationship between m^5^C modifications, amino acid transportation and immune microenvironment. ALYREF might be a novel target for PDAC metabolic vulnerability and immune surveillance.

## Results

### ALYREF is highly expressed in PDAC, and its high expression associates with poor prognosis and immunosuppression

**Fig. 1:**
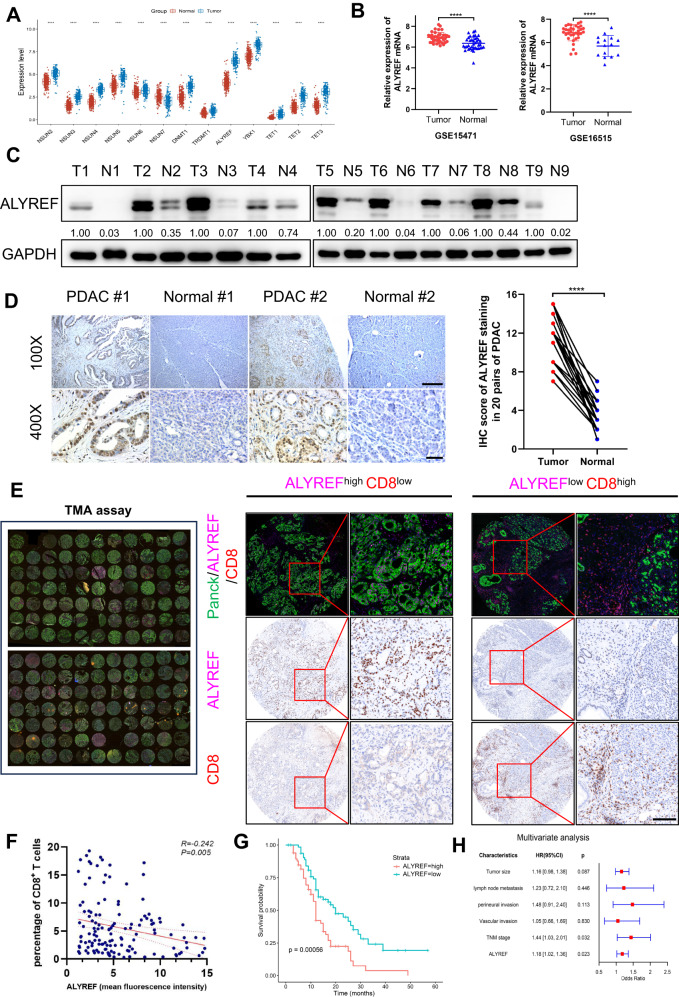
Upregulation of ALYREF in PDAC is associated with poor prognosis and immune escape. **A** The mRNA expression profiles of m^5^C regulators in PDAC and normal tissues were based on TCGA+GTEx databases. **B** The mRNA expression of ALYREF in PDAC and adjacent tissues were obtained from GSE15471 and GSE16515 datasets. **C** ALYREF protein levels were analyzed in PDAC and paired normal tissues by western blotting (n = 9). **D** Representative IHC images and IHC scores of ALYREF staining in 20 pairs PDAC and normal tissues (scale bar, 250 μm (100×), 50 μm (400 ×)). **E**, **F** mIHC was performed on a tissue microarray to detect the relationship between ALYREF and CD8^+^ T cells (*n* = 135, scale bar, 50 μm). **G** Kaplan–Meier curve of overall survival was performed based on the follow-up data of TMA assay. **H** Multivariate analysis of several factors was performed in TMA assay. Data are presented the mean ± SD. ****p < 0.0001.

To explore the role of m^5^C regulators in PDAC, we analyzed the mRNA expression levels of m^5^C regulators in PDAC using TCGA+GTEx databases. All m^5^C regulators were significantly upregulated in PDAC tissues except for NSUN7 which was downregulated in PDAC tissues. Notably, the expression of ALYREF and YBX1 was higher compared with other m^5^C regulators in PDAC. The upregulation of ALYREF was more significant in PDAC tumor tissues compared with normal tissues (Fig. 1A). In addition, ALYREF was significantly upregulated in PDAC tumor tissues in the GSE15471 and GSE16515 datasets (Fig. 1B). ALYREF protein expression was highly upregulated in PDAC tumors compared with the corresponding adjacent non-neoplastic tissues in nine paired samples of tumor tissues and adjacent tissues from The First Affiliated Hospital of Zhejiang University according to western blotting (Fig. 1C). These results were confirmed by immunohistochemical (IHC) staining of 20 pairs of paraffin-embedded tissues (Fig. 1D). Next, through multiplex immunohistochemistry (mIHC) and tissue microarray (TMA), relationship between ALYREF expression and immune microenvironment was evaluated. We observed that CD8^+^ T cells infiltration is negatively correlated with ALYREF expression, although the R value is relatively low (Fig. 1E, F). These results implied that overexpression of ALYREF may shape an immunosuppressive tumor microenvironment. Furthermore, PDAC patients with high ALYREF expression were significantly associated with poor overall survival (OS) based on survival analysis of follow-up data of TMA cohort (Fig. 1G). Multivariate Cox regression analysis also indicated that ALYREF was an independent predictive marker for the prognosis of patients with PDAC (Fig. 1H). These data demonstrated that ALYREF was overexpressed in human PDAC and was closely associated with poor prognosis and immune escape.

### ALYREF knockdown inhibits PDAC proliferation in vitro and in vivo

**Fig. 2:**
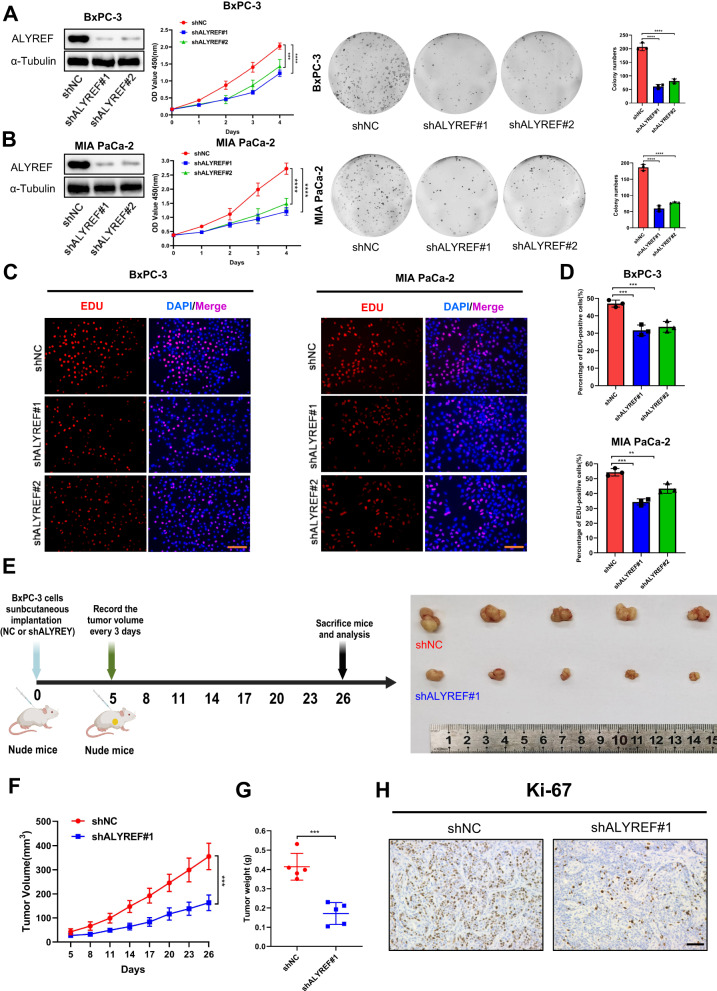
Silencing of ALYREF inhibits PDAC cell proliferation in vitro and in vivo. **A**, **B** Negative control and shRNA (shALYREF #1, #2) were transfected into BxPC-3 and MIA PaCa-2 cells, respectively. The knockdown of efficiency was tested by western blotting and ability of cell proliferation was assessed by CCK-8 and colony formation assay. **C**, **D** Edu assay was used to assess the cell proliferation ability in negative control and shRNA group (scale bar, 100 μm). **E** Schematic protocol of subcutaneous implantation of pretreated BxPC-3 cells (shNC or shALYREF). **F**–**H** Tumor volumes were monitored every two days and tumor were extracted and weighed after 26 days. Representative IHC images of Ki-67 expression (scale bar, 100 μm). Data are presented the mean ± SD of 3 independent experiments. **p < 0.01, ***p < 0.001, ****p < 0.0001.

We knocked down ALYREF in the BxPC-3 and MIA PaCa-2 cell lines to evaluate the biological effects of ALYREF on PDAC. The CCK-8 assays, colony formation assays and Edu assay revealed that ALYREF knockdown significantly reduced cell proliferation in the shALYREF group compared to that in the control group (Fig. 2A–D). A tumor xenograft model was constructed by subcutaneous injection of stable knockdown PDAC cells into nude mice to validate the effect of ALYREF in vivo (Fig. 2E). ALYREF knockdown inhibited tumorigenesis and significantly reduced tumor volume and weight compared to controls (Fig. 2F, G). The IHC results showed that tumor tissue from ALYREF knockdown group has a lower Ki-67 level in vivo (Fig. 2H). These findings confirmed that ALYREF knockdown reduced the proliferation and growth PDAC cells in vitro and in vivo.

### Re-expressing ALYREF restores tumorigenic ability of ALYREF knockdown PDAC cells

**Fig. 3:**
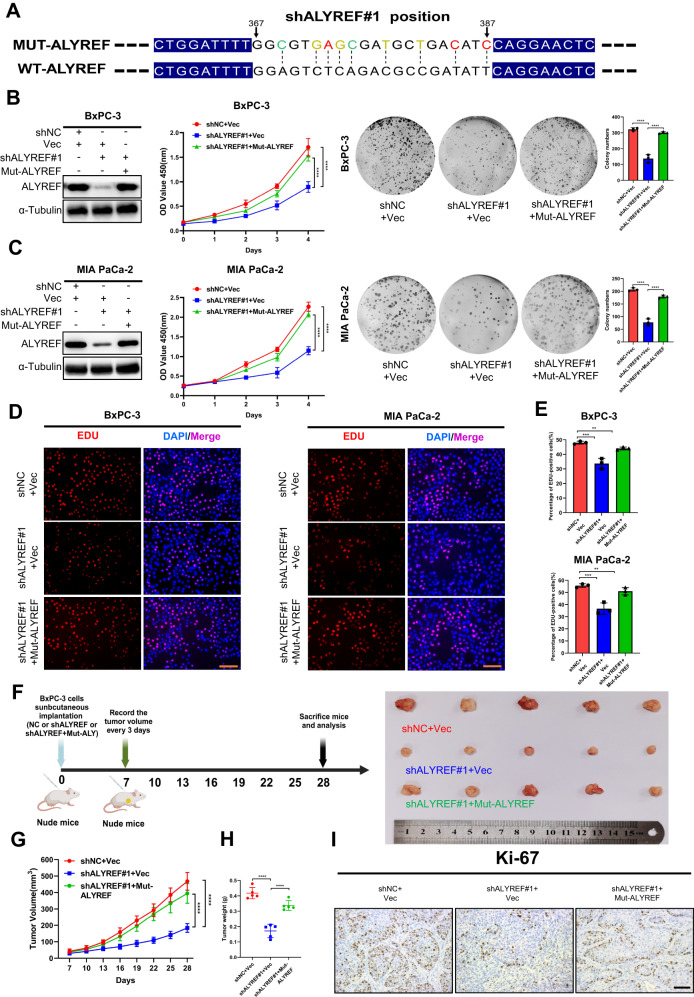
Re-expression of ALYREF can rescue the phenotype induced by ALYREF knockdown. **A** The diagram showed synonymous mutant sites for expression recovery of ALYREF, and mutant bases were marked by different colors. **B**, **C** The protein level of ALYREF were detected by western blotting in ALYREF-knockdown cells after re-expressing ALYREF. CCK-8 and colony formation assay were used to test whether the recovery of ALYREF can alleviate damage to ALYREF knockdown cells in vitro. **D**, **E** Edu assay was used to assess the cell proliferation ability in three groups (scale bar, 100 μm). **F** Schematic protocol of subcutaneous implantation of pretreated BxPC-3 cells (shNC+Vec, shALYREF#1+Vec and shALYREF#1+Mut-ALYREF). **G**–**I** Tumor volumes were monitored every two days and tumor were extracted and weighed after 28 days. Representative IHC images of Ki-67 expression (scale bar, 100 μm). Data are presented the mean ± SD of 3 independent experiments. ** *p* < 0.01, ***p < 0.001, ****p < 0.0001.

ALYREF knockdown can inhibit cell proliferation and affect the growth of PDAC in vivo. However, it is unclear whether restoring ALYREF expression can alleviate the damage to these knockdown cells. Therefore, we constructed the ALYREF overexpression vector which comprised synonymous mutations for the target site of shALYREF#1 (Fig. 3A). Western blotting demonstrated that ALYREF was successfully restored in ALYREF-knockdown PDAC cells. In addition, these inhibitory effects were rescued by re-expression of ALYREF (Fig. 3B–E). Moreover, we generated a subcutaneous xenograft tumor model to investigate whether the recovery of ALYREF expression can rescue tumor growth in vivo (Fig. 3F). Overexpression of mutant ALYREF in ALYREF-depleted BxPC-3 cells remarkably restored tumor weights, tumor volumes and Ki-67 expression level (Fig. 3G–I). Thus, these results implied that ALYREF promotes tumor cells proliferation and tumor growth.

### Targeting ALYREF inhibits PDAC growth partly by enhancing CD8^+^ T cells-mediated antitumor effect

**Fig. 4:**
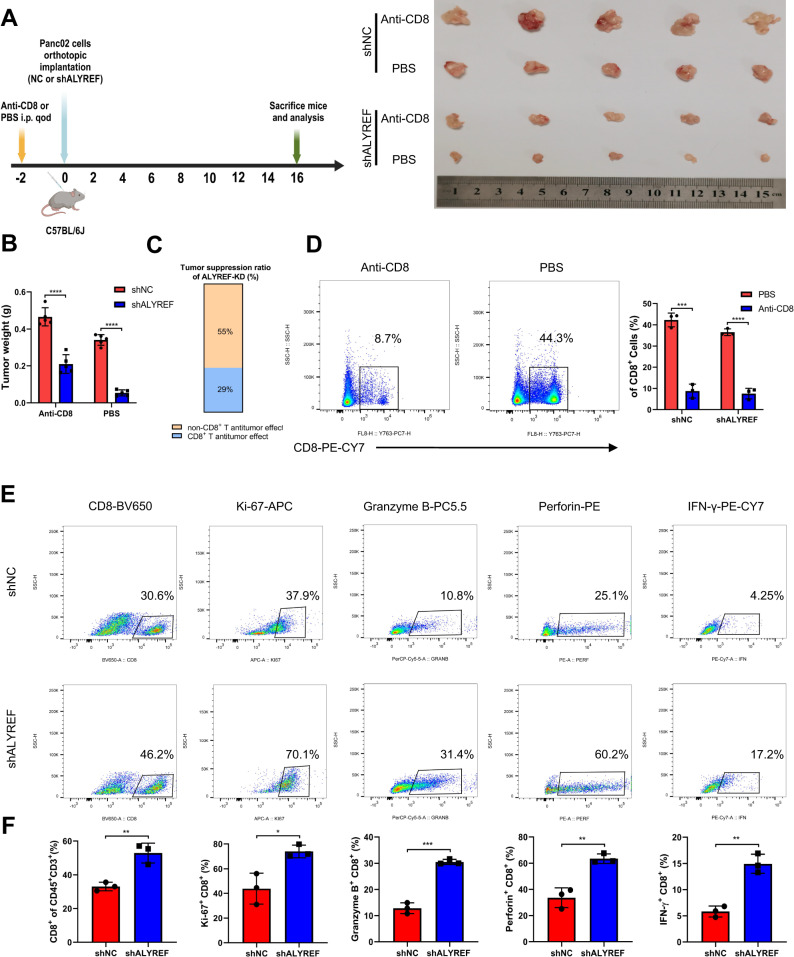
CD8^+^ T cells also play an important role in antitumor effect mediated by ALYREF knockdown. **A** Schematic protocol displaying that shNC or shALYREF panc02 cells were orthotopically injected into normal C57BL/6J and CD8^+^ T cell deficient C57BL/6J mice separately. **B**, **C** Weight of tumor in each group and tumor suppression ratio of ALYREF knockdown was calculated by $1-\frac{m{ean}\,{tumor}\,{weight}({shALYREF})}{m{ean}\,{tumor}\,{weight}\,({shNC})}$ (n = 5). **D** Representative flow cytometry figures and quantification of CD8^+^ T cells staining of splenocytes (n = 3). **E**, **F** Representative flow cytometry figures of CD8^+^ T cells, Ki-67^+^ CD8^+^ T cells, Granzyme B^+^ CD8^+^ T cells, Perforin^+^ CD8^+^ T cells and IFN-γ^+^ CD8^+^ T cells and the statistical analysis of these results (n = 3). Data are *p*resented the mean ± SD of 3 independent experiments. *p < 0.05, **p < 0.01, ***p < 0.001, ****p < 0.0001.

Because of the negative correlation between ALYREF and CD8^+^ T cells infiltration (Fig. 1F), we next explored whether ALYREF have an influence on tumor immune microenvironment. First, we constructed Panc02 cell lines with ALYREF silencing and the knockdown efficiency was test by western blotting (Supplementary Fig. S1). Next, Panc02 cells both with and without ALYREF knockdown were orthotopically implanted into nude and C57BL/6J mice, respectively. Compared to nude mice, ALYREF deficiency resulted in a more significant reduction in tumor weight in C57BL/6J mice, which indicated that ALYREF deficiency could promote antitumor immune response (Supplementary Fig. S2). Moreover, to further assess the influence of ALYREF knockdown on CD8^+^ T cells, we constructed the CD8^+^ T cell deficient C57BL/6J mice using an anti-CD8 mAb. Subsequently, shNC or shALYREF panc02 cells were orthotopically injected in CD8^+^ T cell deficient C57BL/6J and normal C57BL/6J mice respectively (Fig. 4A). The results showed that ALYREF knockdown suppressed tumor burden by approximately 55% compared with control group in CD8^+^ T cell deficient C57BL/6J mice. However, this suppression ration significantly increased to 84% in normal C57BL/6J mice (Fig. 4B, C). The depletion of CD8^+^ T cells was confirmed by flow cytometry (Fig. 4D). Therefore, these results indicated that CD8^+^ T cells played an essential role in antitumor effect induced by ALYREF knockdown. Then, we collected tumor tissues from normal C57BL/6J mice to performed flow cytometry analysis. The results indicated that inhibition of ALYREF drastically strengthened the proliferation and effector function of CD8^+^ T cells (Fig. 4E, F). Taken together, these findings manifested that antitumor effect induced by ALYREF knockdown was partly governed by CD8^+^ T cells.

### ALYREF interference inactivates mTORC1 signaling largely through repressing the expression of SLC7A5

**Fig. 5:**
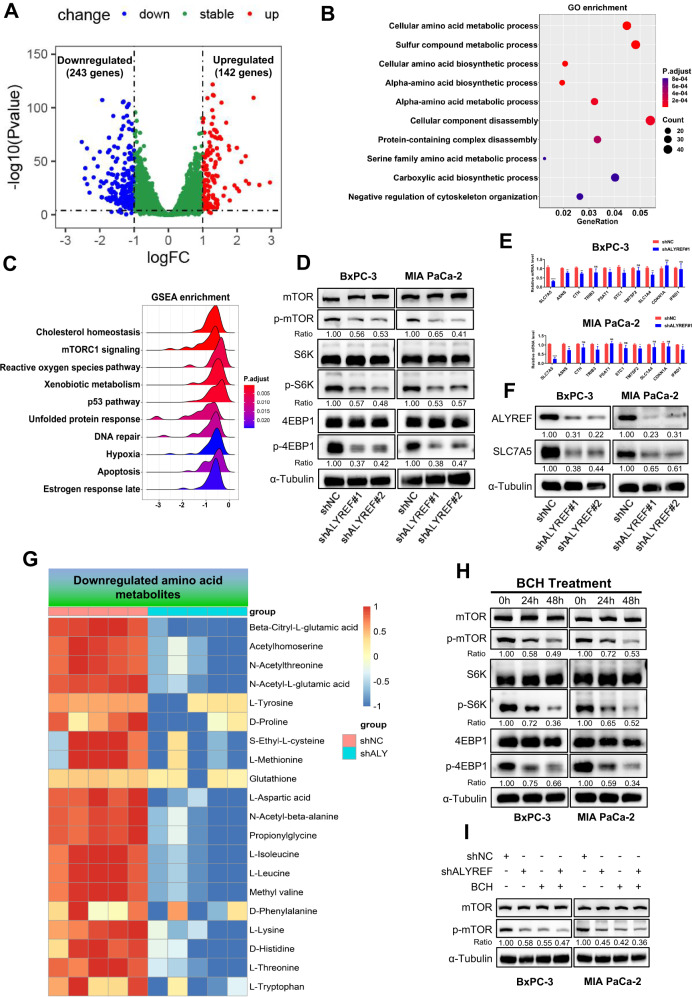
ALYREF knockdown impair mTORC1 signaling through downregulating SLC7A5. **A** RNA-seq was performed on MIA PaCa-2 cells transfected with shALYREF#1 or shNC. The results of differential expression analysis of were presented by volcano plot. **B**, **C** GO and GSEA enrichment analysis for differentially expressed genes and the top ten pathways are shown. **D** Western blotting of mTOR, p-mTOR, S6K, p-S6kK, 4EBP1 and p-4EBP1 levels in shNC or shALYREF cells. **E**, **F** RT-qPCR was conducted on ALYREF knockdown cells to validate top ten genes from mTORC1 signaling of GSEA analysis. The protein level of SLC7A5 was verified by western blotting. **G** Heatmap of LC-MS/MS analysis shows the downregulated amino acid metabolites. **H** After treated with BCH (30 mM) for 0 h, 24 h and 48 h, western blotting of mTOR, p-mTOR, S6K, p-S6kK, 4EBP1 and p-4EBP1 levels were detected. **I** Protein levels of mTOR and p-mTOR were measured by western blotting in the indicated cells. Data are presented the mean ± SD of 3 independent experiments. *p < 0.05, **p < 0.01, ****p < 0.0001, ns no significance.

We conducted RNA-seq in control or ALYREF-knockdown MIA PaCa-2 cells to investigate transcriptional differences and determine the underlying mechanism by which ALYREF knockdown exerts tumor inhibitory effects in PDAC. Silencing of ALYREF differentially expressed 385 mRNAs, including 243 downregulated mRNAs and 142 upregulated mRNAs (Reads ≥100, |Fold change|≥2, P < 0.05) (Fig. 5A). Subsequently, GO and GSEA enrichment analyses of differentially expressed genes showed that some pathways associated with amino acid metabolism and mTORC1 signaling were downregulated (Fig. 5B, C). Thus, we hypothesized that ALYREF knockdown impaired mTORC1 signaling to inhibit the proliferation of PDAC cells. We detected the key proteins involved in mTORC1 signaling in BxPC-3 and MIA PaCa-2 cells after ALYREF knockdown using western blotting. The proportions of phosphorylated mTOR, S6K, and 4EBP-1 were significantly downregulated (Fig. 5D). Next, to identify the key regulator impacting the mTORC1 signaling, we employed RT-qPCR to measure the top 10 candidates of core genes which were enriched in mTORC1 signaling of GSEA analysis. SLC7A5 was identified as the overriding regulator since it showed the most significant and consistent change (Fig. 5E). The differential expression of SLC7A5 in ALYREF-knockdown cells was further verified at the protein level (Fig. 5F). SLC7A5 is a member of the solute carrier superfamily and functions as an essential amino acid transporter. SLC7A5 mainly transports LNAAs, including leucine, isoleucine, valine, phenylalanine, tyrosine, tryptophan, methionine, and histidine. Interestingly, our metabolomics data also showed that tyrosine, methionine, leucine, isoleucine, and histidine were decreased after ALYREF knockdown (Fig. 5G). To validate the effects of SLC7A5 on mTORC1 signaling, we treated BxPC-3 and MIA PaCa-2 cells with BCH (a small molecule inhibitor of SLC7A5). The western blotting results revealed that the phosphorylated level of mTOR, S6K, and 4EBP-1 were downregulated over time (24 h vs. 48 h) (Fig. 5H). In addition, compared to ALYREF knockdown or BCH treatment alone, combination treatment only slightly further inhibits the activity of mTORC1 signaling (Fig. 5I). Therefore, these findings illustrated that ALYREF knockdown dampened mTORC1 signaling mainly by downregulating SLC7A5 expression.

### SLC7A5 is responsible for tumor progression and immune escape derived from ALYREF

**Fig. 6:**
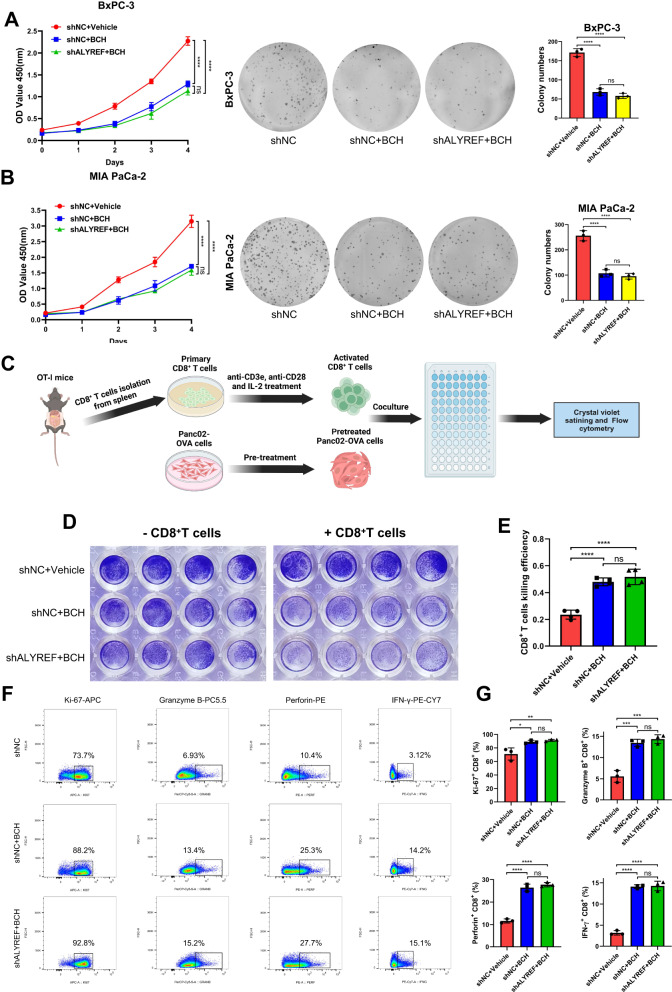
ALYREF promotes tumor progression and immune escape through SLC7A5. **A**, **B** CCK-8 and colony formation were used to evaluate the proliferation ability of the indicated cells. **C** Schematic protocol of tumor cells killing assay mediated by CD8^+^ T cells. **D**, **E** Representative images and statistical result of CD8^+^ T cells cytotoxicity assay (n = 4). CD8^+^ T cells killing efficiency was calculated by $1-\frac{{OD\; value}(-{\rm{CD}}8+{\rm{T}})}{{OD\; value}(+{\rm{CD}}8+{\rm{T}})}$. **F**, **G** Representative flow cytometry images of Ki-67^+^ CD8^+^ T cells, Granzyme B^+^ CD8^+^ T cells, Perforin^+^ CD8^+^ T cells and IFN-γ^+^ CD8^+^ T cells and the statistical analysis of these results (n = 3). Data are presented the mean ± SD of 3 independent experiments. *p < 0.05, **p < 0.01, ***p < 0.001, ****p < 0.0001, ns no significance.

We further verified that whether the oncogenic effect and immune escape derived from ALYREF were mediated by SLC7A5. BxPC-3 cells and MIA PaCa-2 cells were treated with BCH and results showed that SLC7A5 inhibition decreased the proliferation ability of PDAC cells. In addition, compared with BCH group, combination treatment of BCH and ALYREF knockdown did not reduce further proliferation rate (Fig. 6A, B). Next, to investigated whether SLC7A5 inhibition boost the effector function of CD8^+^ T cells, we conducted the CD8^+^ T cells killing assay in vitro (Fig. 6C). After coculture with pretreated Panc02-OVA cells (BCH or shALYREF+BCH), CD8^+^ T cells presented a stronger killing ability than control group and there is no significant difference between BCH and shALYREF+BCH group (Fig. 6D, E). The flow cytometry analysis showed that the level of Ki-67, Granzyme B, Perforin and IFN-γ of CD8^+^ T cells from BCH group were significantly increased compared with that from control group. However, this difference was not observed between BCH group and shALYREF+BCH group (Fig. 6F, G). Together, these results suggested that PDAC progression and immune escape induced by ALYREF were depended on SLC7A5.

### ALYREF recognizes m^5^C modifications of JunD which serves as a transcription factor of SLC7A5

**Fig. 7:**
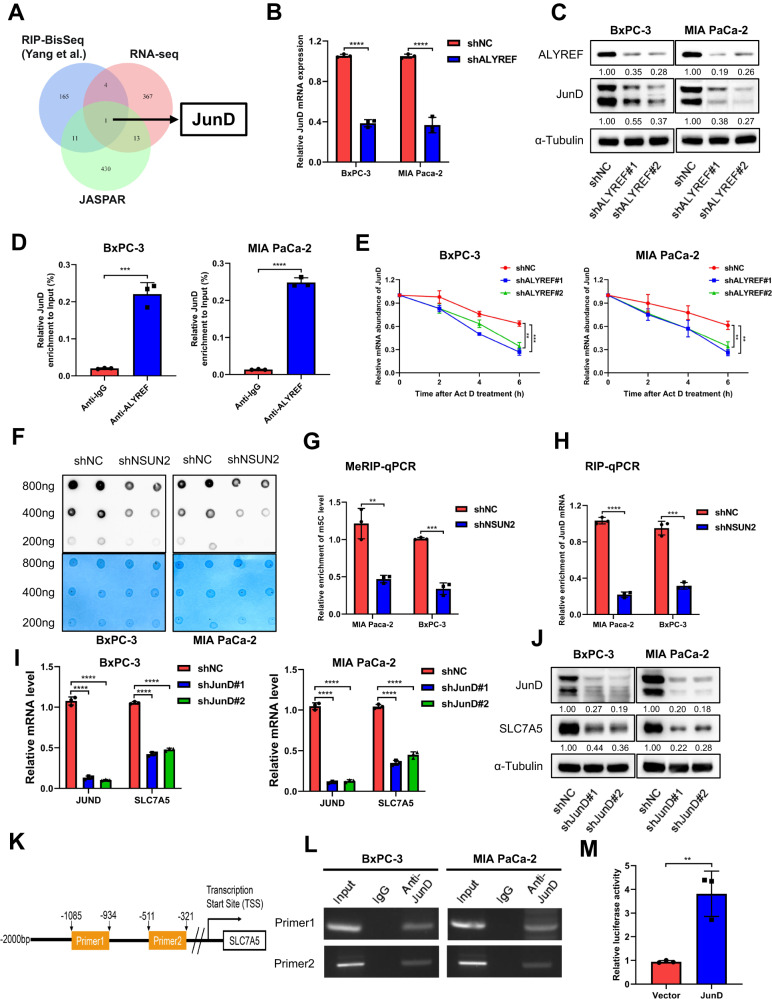
ALYREF directly targeted JunD which promote the transcription of SLC7A5 mRNA. **A** Venn Diagram showing the intersection of RIP-BisSeq, RNA-seq and JASPAR database. JunD is identified as a potential target. **B**, **C** Expression of JunD following ALYREF knockdown was detected by RT-qPCR and western blotting. **D** RIP assay was used to validate the direct binding between ALYREF and JunD. **E** The mRNA decay rate of JunD in shNC and shALYREF PDAC cells treated with Actinomycin D. **F** The dot blot assay was used to assess the change of mRNA m^5^C level after NSUN2 knockdown in PDAC cells. **G** MeRIP-qPCR assay was conducted using m^5^C-specific antibody to measure the m^5^C levels. **H** RIP-qPCR assay was performed using anti-ALYREF antibody to determine the binging efficiency between ALYREF and JunD mRNA. **I**, **J** Expression of SLC7A5 following JunD knockdown was detected by RT-qPCR and western blotting. **K** Schematic illustration showing the position of ChIP- qPCR primers. **L**, **M** ChIP assay was performed to test whether JunD could bind to the promoter of SLC7A5. Dual-luciferase assays verified that whether JunD could promote the transcription of SLC7A5 mRNA. Data are presented the mean ± SD of 3 independent experiments, **p < 0.01, ***p < 0.001, ****p < 0.0001.

To investigate how ALYREF regulates SLC7A5, we explored the ALYREF-RIP-BisSeq data from Yang et al. [12] and found two potential m^5^C sites on SLC7A5. Next, we performed RIP-qPCR with a specific ALYREF antibody. However, there was no significant difference between the IP and IgG groups (Supplementary Fig. S3). This indicated that ALYREF may not directly targeted SLC7A5 in PDAC cells. So, we reasoned that the downregulation of SLC7A5 upon ALYREF knockdown might be attributed to repression of some transcription factors. By intersecting the RIP-BisSeq from Yang et al., our RNA-seq and JASPAR database, JunD was identified as a candidate. (Fig. 7A). Results of RT-qPCR and western blotting demonstrated that JunD was consistently downregulated after ALYREF silencing in BxPC-3 and MIA PaCa-2 cells (Fig. 7B, C). To validate the direct binding between ALYREF and JunD, we designed a specific primer according to the potential m^5^C sites and conducted the RIP-qPCR assays. The RIP-qPCR results verified that JunD is the authentic downstream of ALYREF, and its m^5^C modification can be specifically recognized by ALYREF (Fig. 7D). In addition, the stability of JunD significantly decreased following ALYREF knockdown after actinomycin D treatment (Fig. 7E). Huang et al. discovered that m^5^C modification of mRNA in HeLa cells is mainly catalyzed by NSUN2 [13]. We speculated that similar regulatory mechanism might exist in PDAC cells. NSUN2 was knocked down in BxPC-3 cells and MIA PaCa-2 cells using a short hairpin RNA (Supplementary Fig. S4). Then we tested the m^5^C levels of total mRNA in NSUN2- knockdown BxPC-3 and MIA PaCa-2 cells using dot plot assay. Unsurprisingly, the m^5^C levels of total mRNA were significantly decreased in NSUN2 knockdown samples (Fig. 7F). In addition, we observed that the m^5^C levels of JunD mRNA and the binding efficiency between ALYREF and JunD mRNA were also reduced after NSUN2 knockdown (Fig. 7G, H). These findings proved that ALYREF specifically recognize the m^5^C modification catalyzed by NSUN2.

To verify whether JunD transcriptionally activates SLC7A5, we knocked down JunD using two short hairpin RNAs (shJunD#1 and shJunD#2) in BxPC-3 and MIA PaCa-2 cells. RT-qPCR and western blotting results indicated that JunD knockdown in PDAC cells downregulated SCL7A5 expression (Fig. 7I, J). Chromatin immunoprecipitation (ChIP) assays in PDAC cells using two pairs of primers covering potential JunD-binding sites showed that JunD binds to both sites (Fig. 7K, L). Dual-luciferase reporter assay further confirmed that JunD can stimulate the expression of SLC7A5 (Fig. 7M). Thus, our results demonstrated that JunD was directly regulated by ALYREF in an m^5^C manner and transcriptionally activated SLC7A5.

### The clinical significance of ALYREF-JunD-SLC7A5 axis

**Fig. 8:**
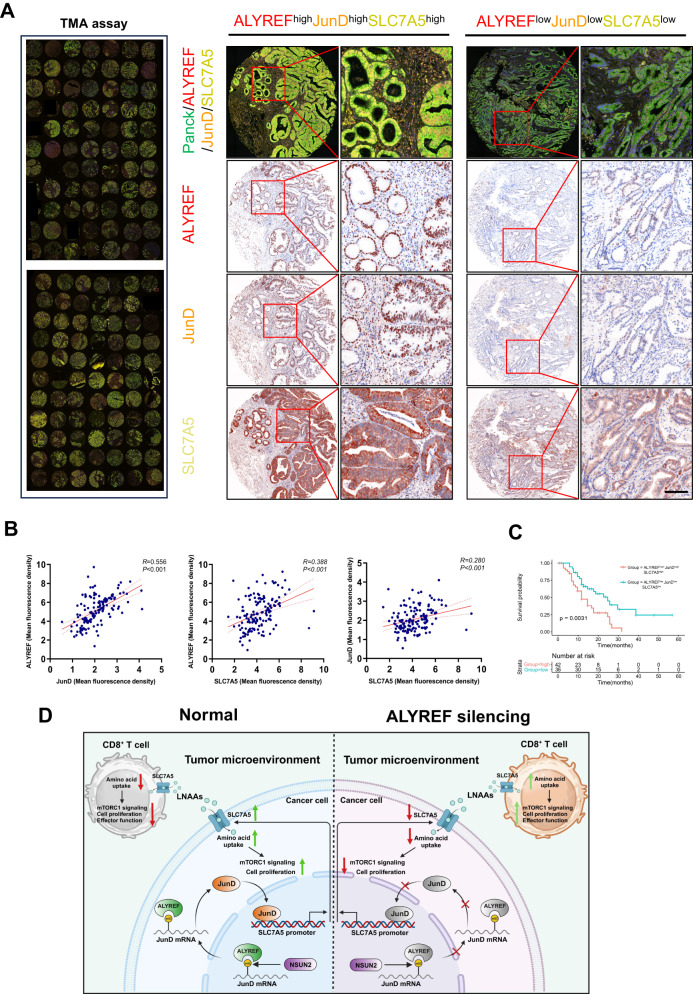
The correlation among ALYREF, JunD and SLC7A5 and the clinical significance of ALYREF-JunD-SLC7A5 axis. **A** The expression levels of ALYREF, JunD and SLC7A5 in TMA were measured by multiplex immunohistochemical and two groups of representative multiplexes immunohistochemical images are shown (scale bar, 50 μm). **B** The correlation of among the expression levels of ALYREF, JunD and SLC7A5 in TMA samples was analyzed by Pearson correlation coefficient (*n* = 139). **C** Overall survival analysis based on TMA cohort showed that patients with high co-expression of ALYREF, JunD and SLC7A5 have poor prognosis. **D** The graphic illustration of ALYREF modulating tumor progression and immune escape in PDAC.

The clinical significance of the ALYREF-JunD-SLC7A5 axis in PDAC progression was assessed by performing a multiplex immunohistochemical assay using a PDAC tissue microarray to evaluate the expression levels of the three proteins (Fig. 8A). ALYREF expression positively correlated with that of JunD and SLC7A5, with correlation coefficients of 0.556 and 0.388, respectively. In addition, JunD expression positively correlated with SLC7A5 expression, with a correlation coefficient of 0.28 (Fig. 8B). Patients with increased co-expression of ALYREF, JunD, and SLC7A5 had a poorer prognosis based on Kaplan-Meier analysis (Fig. 8C). In summary, ALYREF, JunD, and SLC7A5 levels were positively correlated in clinical samples, and the ALYREF-JunD-SLC7A5 axis may be a promising target for therapy and prognosis.

## Discussion

As a research hotspot, modification of mRNA has been receiving numerous attentions in recent years [14–16]. Growing evidence demonstrated that modification of mRNA plays a significant role in various types of cancers [17–19]. Our study found that ALYREF shows the most significant differences between tumor samples and normal tissues and the overexpression of ALYREF is a marker of bad prognosis. Integrated analysis of RIP-BisSeq (Yang et al.), JASPAR database and RNA-seq data revealed that JunD is directly regulated by ALYREF in an m^5^C manner and sustains its expression. The enhanced JunD subsequently activates the transcription of SLC7A5 which promotes the tumor cells proliferation and avoids immune attack (Fig. 8D).

ALYREF (also named THOC4) is the first identified m^5^C reader and mainly located in the nucleus. ALYREF mostly binds the 3′ and 5′ region of mRNA and mediates the export, stabilization and splicing of mRNA [20, 21]. Silencing of ALYREF in PDAC cells significantly inhibited cell proliferation in vitro and decreased tumor growth in vivo. These effects could be rescued by the recovery of ALYREF expression with a synonymous mutation vector. Moreover, mIHC results based on TMA cohort showed that there is a negative correlation between ALYREF expression and CD8^+^ T cells infiltration. The growth suppression of tumor induced by ALYREF knockdown was expanded in normal C57BL/6J compared with CD8^+^ T cell deficient C57BL/6J mice. And flow cytometry analysis suggested that the proliferation ability and effector function of CD8^+^ T cells were drastically enhanced after ALYREF knockdown. These results demonstrated that ALYREF accelerates the PDAC cells proliferation and shapes an immunosuppressive microenvironment.

Many types of tumor cells present different metabolic features compared with non-malignant cells to meet the high demand for nutrition induced by rapid proliferation. Thus, metabolic reprogramming is considered a hallmark of cancer [22]. Aerobic glycolysis (also known as the Warburg effect) is the most renowned metabolic alteration in cancer cells, which results in increased glucose consumption and lactate emission, even in the presence of sufficient oxygen. With substantial advances in our understanding of the mechanisms and biological functions of metabolic reprogramming in cancer, the important role of amino acids in tumor metabolism has received increasing attention. SLC7A5 belongs to the solute carrier superfamily and mainly transports LNAAs, including leucine, isoleucine, valine, phenylalanine, tyrosine, tryptophan, methionine, and histidine [23]. Several studies showed that SLC7A5 presents a high tumor-specific expression in many types of cancer [24–26]. According to previous studies, SLC7A5 interference can inhibit breast cancer growth [27] and promote CD8^+^ T cells function in tumor microenvironment of colorectal cancer [28]. Moreover, Ueda et al. reported that anti-SLC7A5 mAb inhibits colon cancer growth in xenograft model [29]. In our study, GO and GSEA analysis of RNA-seq results showed that multiple amino acid metabolic processes and mTORC1 activity were remarkably impaired in ALYREF knockdown cells. Furthermore, we tested the top 10 genes from mTORC1 signaling of GSEA and results showed that SLC7A5 was the most significantly downregulated. Thus, we speculate that the downregulation of SLC7A5 was the main reason for mTORC1 inactivation caused by ALYREF silencing. Analysis of LC-MS/MS and SLC7A5 inhibitor further demonstrated that ALYREF regulates mTORC1 pathway largely through SLC7A5. In addition, because of the high expression of SLC7A5, PDAC cells outcompete CD8^+^ T cells for LNAAs to support itself growth and simultaneously weaken the activity of CD8^+^ T cells. Our results also manifested that BCH treatment inhibits the proliferation of PDAC cells and enhances the effector function of CD8^+^ T cells. And the combination of BCH and shALYREF produced a same effect with BCH treatment alone. Therefore, SLC7A5 is responsible for tumor progression and immune escape driven by ALYREF.

The dysregulation of transcription factors leads to many diseases, including cancer. MYC is a master regulator of gene transcription that is one of the most frequently mutated oncogenes that drives a variety of biological programs to sustain tumor growth [30]. AP-1 transcriptional complexes consisting of Jun, Fos, ATF, and MAF family members facilitate cancer development [31, 32]. For example, a recent study showed that JunD binds to a subgroup of enhancers to activate the expression of several genes relevant to chemoresistance in PDAC [33]. Moreover, emerging evidence indicates that these oncogenic transcription factors are strongly associated with the metabolic reprogramming of cancer cells. c-Jun, JunB, and C/EBPβ promote glycolysis in melanoma cells through transcriptionally activate genes for glycolysis enzymes and consequentially weaken the anticancer effect of CD8^+^ T cells [34]. In this study, we found that ALYREF binds the m^5^C sites catalyzed by NSUN2 to maintain the stabilization of JunD mRNA. And JunD further activates the expression of SLC7A5 through serving as its transcription factor. Therefore, the identification of ALYREF-JunD-SLC7A5 axis provides a new regulator mechanism to better understand the relationship between epitranscriptome and metabolic reprogramming.

In summary, our study illustrates the clinical significance of ALYREF and its oncogenic role in human PDAC. ALYREF knockdown inhibits PDAC cells proliferation and attenuates tumor immune escape by impairing the JunD-SLC7A5-mTORC1 axis. These findings provide new insights into developing potential therapeutic strategies for PDAC.

## Materials and methods

### Patients and specimens

A total of 29 human PDAC tissues and adjacent normal tissues were obtained from the Department of Hepatobiliary and Pancreatic Surgery, the First Affiliated Hospital, School of Medicine, Zhejiang University. Nine pairs of frozen tissues were subjected to protein extraction for western blotting analysis and 20 pairs of paraffin-embedded tissues were applied for immunohistochemistry analysis. The human PDAC tissue microarray was created by Wuhan Servicebio technology (Wuhan, China) using 156 PDAC tissue specimens from the First Affiliated Hospital, School of Medicine, Zhejiang University, China. This study was approved by the Institutional Review Board at the First Affiliated Hospital, School of Medicine, Zhejiang University.

### Cell culture

BxPC-3, MIA PaCa-2, 293T and Panc02 cell lines were purchased from the American Type Culture Collection (ATCC, Manassas, VA, USA). MIA PaCa-2, 293T and Panc02 cells were grown in high-glucose Dulbecco’s modified Eagle’s medium (DMEM, Hyclone, UT, USA) containing 10% fetal bovine serum (Thermo Fisher Scientific) and 1% penicillin and streptomycin (Cienry, Hangzhou, China). BxPC-3 cells were grown in RPMI-1640 medium (Hyclone, UT, USA) supplemented with 10% fetal bovine serum (Thermo Fisher Scientific, MA, USA) and 1% penicillin and streptomycin (Cienry, Hangzhou, China). All cells were cultured in at 37 °C in a humidified 5% CO_2_ incubator. Cells were routinely tested for mycoplasma contamination.

### Plasmid construction and cell transfection

The shRNA fragments targeting ALYREF (human and mouse), JunD and NSUN2 were cloned into the pLKO.1 vector (Shhebio, Shanghai, China). The sequences of synonymous mutation of ALYREF were produced by overlap extension PCR and cloned into pLVX-EF1a-IRES-mCherry plasmid (Addgene, Watertown, MA, USA). The lentiviral plasmid pLV3-CMV-OVAL (chicken)-CopGFP-Puro (Addgene, Watertown, MA, USA) was used to overexpress the ovalbumin (OVA). For lentivirus production, 4 µg of targeted plasmids, 2 µg of plasmid psPAX2, and 1 µg of plasmid pMD2.G were added to 500 µL of jetPRIME buffer with 4 µL jetPRIME reagent (Polyplus, Illkirch, France) for 15 min, and subsequently added to 293T cells in a 10 cm dish. After 48 h, the supernatant was filtered through a 0.45-μm Stericup filter unit (Millipore, MA, USA) and concentrated using a Universal Virus Concentration Kit (Beyotime Biotechnology, Shanghai, China). Cells were grown until 50–60% confluence, then transfected with the lentivirus for 24 h. Puromycin (2 mg/mL) was used to select stably transfected cells. All target sequences and primers are listed in Supplementary Table S1.

### Western blotting and antibodies

Total protein was extracted from cells or tissues using RIPA buffer (Beyotime Biotechnology, Shanghai, China) containing a protease inhibitor cocktail and phosphatase inhibitor cocktail (Bimake, TX, USA) for 30 min on ice. The samples were centrifuged at 12,000 × *g* for 15 min, the supernatant was collected, and the protein concentration was determined using a bicinchoninic acid (BCA) protein assay (Beyotime Biotechnology, Shanghai, China). The protein samples were separated by SDS-PAGE, transferred to 0.45-μm polyvinylidene difluoride (PVDF) membranes (Millipore, USA), blocked using 5% skim milk for 1 h, and the membranes were incubated with the corresponding primary antibodies at 4 °C overnight. The membranes were washed thrice with TBST and incubated with secondary antibodies for 1 h at room temperature. The bands were visualized using an enhanced chemiluminescence (ECL) detection buffer (Vazyme, Nanjing, China). The primary antibodies used for western blotting were as follows: anti-α-tubulin (Proteintech Group, Rosemont, IL, USA; 66031-1-Ig; 1:5000), anti-ALYREF (Cell Signaling Technology, Danvers, MA, USA; 12655; 1:1000), anti-SLC7A5 (Cell Signaling Technology, Danvers, MA, USA; 32683; 1:1000), anti-JunD (Cell Signaling Technology, Danvers, MA, USA; 5000; 1:1000), mTOR substrates antibody sampler kit (Cell Signaling Technology, Danvers, MA, USA; 9862; 1:1000), anti-P70 S6 kinase (Cell Signaling Technology, Danvers, MA, USA; 34475; 1:1000), and anti-4EBP1 (Cell Signaling Technology, Danvers, MA, USA; 9644; 1:1000), anti-NSUN2 (Proteintech, Rosemont, IL, USA; 20854-1-AP; 1:5000).

### Immunohistochemistry

Paraffin-embedded tissues were cut into 4 μm-thick sections and baked for 60 min at 68 °C. The section was deparaffinized, rehydrated, then pretreated using heat-mediated antigen retrieval with sodium citrate buffer (pH 6), then blocked using 3% bovine serum albumin (BSA). Sections were incubated overnight at 4 °C with antibody against ALYREF (Cell Signaling Technology, Danvers, MA, USA; 12655; 1:200) or Ki-67 (Abcam, Cambridge, MA, USA; ab16667, 1:200). Next day, sections were incubated with horseradish peroxidase (HRP)-conjugated secondary antibody for 1 h at room temperature. Signals were amplified using a diaminobenzidine (DAB) chromogen kit (BDB2004, Biocare, Redditch, UK) and the nuclei were stained with hematoxylin. Images of representative fields were obtained using ImageScope software (Leica Biosystems). IHC scores of ALYREF were evaluated using a semi-quantitative system based on intensity grade and positive percentage. The intensity grade was divided into 0, 1, 2, or 3, implying negative, weakly positive, moderately positive, or strongly positive, respectively. The positive rate score was defined according to the positive area: 0 = 0%, 1 = 1–25%, 2 = 26–50%, 3 = 51–75%, and 4 = 75%.

### Cell proliferation assay and colony formation

Cell proliferation was evaluated using cell counting kit-8 (CCK-8) (GLPBIO, Montclair, CA, USA; GK10001). Two thousand cells per well were seeded in a 96-well plate with five replicates. Culture medium was replaced with fresh complete medium containing CCK-8 (1:9) and cells were incubated at 37 °C for 1 h before detecting the absorbance at 450 nm. The optical density was measured daily at 450 nm for four consecutive days. Colony formation assays involved seeding 1000 treated cells into a 6-well dish and culturing for 10–12 days. The cells were washed with PBS three times, fixed with 4% paraformaldehyde for 20 min, and stained with a 0.1% crystal violet solution. Finally, the plates were photographed, and the colony numbers counted.

### EdU assay

Pre-treated cells were seeded into 6-well plates and cultured for 12 h. After adhesion, EdU was added into medium and incubated for 2 h at a concentration of 5 μm. Then discarding supernatant, cells were fixed with 4% paraformaldehyde for 15 min and permeated by PBS containing 0.3% Triton X-100 for 10 min. BeyoClick™ EdU Cell Proliferation Kit with Alexa Fluor 555 (Beyotime Biotechnology, Shanghai, China) was employed to EdU staining according to manufacturer’s instructions and then 6-well plates was photographed on fluorescence microscope.

### CD8^+^ T cell isolation and cytotoxic T lymphocyte killing assay

Primary CD8^+^ T cells were isolated from the spleen of OT-I mouse. After being sacrificed, the spleen was extracted and pestled on a 40-μM filter to make single-suspension. After depleting red blood cells, CD8^+^ T cells were sorted using a CD8a+ T cell isolation kit (Miltenyi Biotec, Bergisch Gladbach, Germany; 130-104-075). Then the isolated CD8^+^ T cells were cultured with RPMI-1640 complete medium which contained anti-CD3e (BioLegend, 100340, 1 mg/mL), anti-CD28 (BioLegend, 102116, 0.5 mg/mL) and Recombinant mouse IL-2 (MCE, HY-P7077, 100 IU/mL). After 72 h, activated CD8^+^ T cells were co-coltured with pretreated Panc02-OVA cells in 96-well plates (CD8^+^ T cell number/Panc02-OVA cell number = 10:1). Two days later, CD8^+^ T cells were collected for flow cytometry analysis. The remaining tumor cells were fixed with 4% paraformaldehyde, stained with a 0.1% crystal violet solution and photographed. Thereafter, crystal violet was dissolved by 33% acetic acid and subsequently detected the absorbance at 450 nm.

### Animal experiment

All the animal experiments were approved by the Ethics Committee for Laboratory Animals of the First Affiliated Hospital of Zhejiang University, China. For subcutaneous xenograft model, 2 × 10^6^ stably transfected BxPC-3 cells in 100 μL PBS were subcutaneously implanted into right flank of BALB/c nude mice (male, 4-5 weeks old). The length and width of the tumor were measured with calipers every 3 d, and tumor volume was calculated using the following formula: 0.5 × Length × Width^2^. Mice were sacrificed at the end of the feeding period (26 or 28 days), and the subcutaneous tumors were collected, weighed, photographed and immunohistochemistry. For the orthotopic tumor model, 1 × 10^5^ stably transfected Panc02 cells in 25 μl of PBS was injected into the pancreas of C57BL/6 under anesthesia. Drug treatment as follow: Anti-CD8 monoclonal antibody (Bio X Cell, YTS 169.4, 100 mg/mouse, i.p., qod). 16 days after injection, the mice were sacrificed, and the tumors were collected for weighing and further analysis. All the data of tumor volume and weight was normalized over animal weight.

### Flow cytometry

Tumor tissues were cut up and incubated with digest buffer (DMEM medium with 2% FBS, collagenase IV (Thermo Fisher Scientific, 17104019, 1 mg/ml) and DNase (Sigma, D5025, 10 μg/ml)) at 37 °C for 30 min. Then tumor tissues were filtered through 70 μm Cell Strainers (Sigma, CLS431751-50EA) to make sinle-cell suspensions. Cocultured cells in vitro did not need the process above. The Fixable Violet Dead Cell Staining Kit (Thermo Fisher Scientific, L34960, BV421) was applied to exclude dead cells. Cells were incubated with TruStain FcX™ antibody (Biolegend, San Diego, CA, USA) to block non-specific binding and cell surface markers were stained. For intracellular markers, cells were fixed and permeabilized using eBioscience™ forkhead box O3 (FOXP3)/Transcription Factor Staining Buffer Set (Thermo Fisher Scientific, 00-5523-00). Data were collected on Fortessa flow cytometer (Becton Dickinson, Franklin Lakes, NJ, USA) and analyzed using FlowJo software (Becton Dickinson, version 10.8.1). Antibodies used for flow cytometry were as follow: Brilliant Violet 785 anti-mouse CD45 antibodies (Biolegend, 103111), Fluorescein isothiocyanate (FITC)-anti-mouse CD3 antibodies (Biolegend, 100203), Phycoerythrin (PE)/cyanine-7 (Cy7) anti-mouse CD8a antibodies (Biolegend, 100722), Peridinin chlorophyll protein complex (PerCP)/Cyanine5.5 anti-human/mouse Granzyme B recombinant antibodies (Biolegend, 372212), Allophycocyanin (APC) anti-mouse Ki-67 antibodies (Biolegend, 652405), Phycoerythrin (PE) anti-mouse Perforin antibody (Biolegend, 154305), Brilliant Violet 605 anti-mouse IFN-γ antibodies (Biolegend, 505839).

### RNA extraction and quantitative RT-PCR

Total RNA was isolated using an RNA-Quick Purification Kit (YiShan biotechnology, Shanghai, China; RN-001) and reverse transcribed with a HiScript III 1st Strand cDNA Synthesis Kit (Vazyme, Nanjing, China; R312-01). Quantitative real-time PCR analysis was performed using ChamQ SYBR qPCR Master Mix (Vazyme, Nanjing, China; Q321-02) in a real-time PCR system (Applied Biosystems, CA, USA). The relative RNA expression levels were calculated by the 2^ΔΔCt^ method, with the levels normalized to ACTB mRNA. The primers used in this study are listed in Supplementary Table S1.

### RNA sequencing

mRNA sequencing was performed on ALYREF-knockdown (shALYREF#1) and control (shNC) MIA PaCa-2 cells. Total RNA was extracted using the RNA-Quick Purification Kit (YiShan Biotechnology, Shanghai, China; RN-001). Library construction, RNA sequencing (Illumina, San Diego, CA, USA), and analyses were performed at the Biomedical Big Data Center of the First Affiliated Hospital, School of Medicine, Zhejiang University. Raw data that passed the quality control were subjected to differential expression and functional analyses.

### Metabolite analysis by LC–MS/MS

ALYREF-knockdown (shALYREF#1) and control (shNC) MIA PaCa-2 cells were used to metabolite analysis. LC-MS/MS analysis was performed by Mass Spectrometry Detection Platform of the First Affiliated Hospital, School of Medicine, Zhejiang University. Briefly, transfected cells were collected and lysed with 500μl of pre-cooled acetonitrile/water solution (4:1, v/v). After vortex and sonication, the lysate was incubated at −20 °C for 10 min. Then centrifuged at 14,000 × *g* for 15 min, the supernatant was concentrated to dry in a vacuum. Resuspend the samples with acetonitrile/water solution (1:1, v/v), centrifuged at 14,000 × *g* for 15 min, the supernatant was used to LC-MS/MS analysis. The chromatographic separation was performed with a Shimadzu LC-40 UPLC System. After separated by an ultra-high performance liquid chromatography system and injected into an ESI ion source for ionization, the samples were analyzed by a ZenoTOF 7600 mass spectrometer. The raw data was aligned, retention time corrected, and peak area extracted using SCIEX OS 3.1 software. The extracted data is first subjected to metabolite structure identification, data preprocessing, experimental data quality evaluation, and finally data analysis.

### BCH (LAT1-IN-1) treatment

BCH (LAT1-IN-1) (MedChemExpress, NJ, USA; HY-108540) was purchased as a selective and competitive inhibitor of SLC7A5 to explore the biological function of SLC7A5 in PDAC cells. MIA PaCa-2 and BxPC-3 cells were treated with 30 mM BCH for 24 or 48 h to determine mTOR pathway activity using western blotting. MIA PaCa-2 and BxPC-3 cells were treated with BCH at a final concentration of 10 mM and then cells proliferation ability was evaluated by CCK-8 assay and colony formation. Panc02 cells were pre-treated with 30 mM BCH for 48 h and then co-cultured with CD8^+^ T cells.

### RNA immunoprecipitation (RIP) assay

A Magna RIP Kit (Millipore, MA, USA; 17-700) was used to perform the RIP assay following the manufacturer’s instructions. More than 2 × 10^7^ cells were collected and resuspended in the lysis buffer. Magnetic beads were mixed with anti-ALYREF (Cell Signaling Technology, Danvers, MA, USA; 12655; 1:200) and rabbit IgG (Millipore, MA, USA; PP64B; 5 μg) and incubated with rotation for 30 min at room temperature. Then RIP lysate was added to the bead-antibody complex in RIP immunoprecipitation buffer and incubated with rotating overnight at 4 °C. The beads were rinsed with RIP wash buffer five times and incubated with Proteinase K buffer at 55 °C to digest protein. RNA was purified using RNA extraction reagent (ACMEC, Shanghai, China; AC13321) and analyzed by RT-qPCR. The RIP primers are listed in Supplementary Table S1.

### RNA stability assay

RNA decay experiments were performed to assess RNA stability. Briefly, stable transfected cells were treated with Actinomycin D (MedChemExpress, NJ, USA; HY-17559) at a final concentration of 5 μg/mL. The cells were harvested after 0, 2, 4, and 6 h. Total RNA was isolated and subjected to RT-qPCR to quantify the relative expression of JunD mRNA.

### RNA m^5^C dot blotting

Total RNA was isolated as described above and mRNA purification was performed with BeyoMag™ mRNA Purification Kit with Magnetic Beads (Beyotime Biotechnology, Shanghai, China; R0071S) according to manufacturer’s instructions. The concentration of mRNA samples was diluted to 400, 200 and 100 ng/μl. After denatured at 95 °C for 5 min, 2ul of sample of different concentrations were loaded to a nylon membrane (Beyotime Biotechnology, Shanghai, China; FFN10). The membrane was crosslinked by ultraviolet and blocked with non-fat milk. Then the membrane was incubated with m^5^C antibody (Cell Signaling Technology, Danvers, MA, USA; 28692; 1:1000) overnight at 4 °C. Next day, after incubated with secondary antibodies for 1 h at room temperature, the memberane was visualized by a chemiluminescence system (Clinx). Last, the membrane was stained with methylene blue staining buffer (0.2% methylene blue in 0.4 M sodium acetate and 0.4 M acetic acid) for a loading control.

### Methylated RNA immunoprecipitation (MeRIP) assay

A m^5^C MeRIP kit (BersinBio, Guangzhou, China; Bes5204-2) was used to perform the MeRIP assay according to the manufacturer’s instructions. In short, total RNA was isolated from 2 × 10^7^ cells and randomly fragmented into 200 nt. 1/10 volume of fragmented RNA sample was saved as “Input”. After incubated with 5ug of m^5^C antibody or 5ug IgG antibody at 4 °C for 4 h, the immunoprecipitation complex was incubated with protein A/G magnetic beads at 4 °C for 1 h. Then magnetic beads were washed 3 times and incubated with Proteinase K buffer at 55 °C for 30 min. RNA was purified using RNA extraction reagent (ACMEC, Shanghai, China; AC13308) and analyzed by RT-qPCR with normalization to the Input.

### Dual-luciferase reporter assays

Five plasmids (pGL4.10, pGL4.10-SLC7A5 promoter, pcDNA3.1(+)-MCS-3×FLAG, pcDNA3.1(+)-JunD-3xFLAG, and pRL-CMV) were purchased from Obio Technology Corp (Shanghai, China) for the interaction of JunD and SLC7A5-promotor experiment. HEK 293T cells were randomly divided into the following four groups: pGL4.10+ pcDNA3.1(+), pGL4.10+ pcDNA3.1(+)-JunD, pGL4.10-SLC7A5 promoter+pcDNA3.1(+), and pGL4.10-SLC7A5 promoter+pcDNA3.1(+)-JunD. Four groups of cells were transfected with the corresponding plasmids and the pRL-CMV plasmid for 48 h. The cells were collected to calculate the ratio of firefly to Renilla luciferase activity using the Dual Luciferase Reporter Gene Assay Kit (Beyotime Biotechnology, Shanghai, China; RG027).

### Chromatin immunoprecipitation (ChIP) assay

MIA PaCa-2 and BxPC-3 cells were used to conducted to CHiP assays using the Chromatin Immunoprecipitation (ChIP) Assay Kit (Beyotime Biotechnology, Shanghai, China; P2078). A total of 10^7^ cells were cross-linked with 37% formaldehyde for 10 min at 37 °C and then neutralized using glycine. The cells were collected and treated with SDS lysis buffer containing 1 mM phenylmethylsulphonyl fluoride (PMSF) (Beyotime Biotechnology, Shanghai, China; ST506). Cell lysates were fragmented to 200–1000 bp by ultrasonication and mixed with ChIP dilution buffer. Lysates were incubated with anti-JunD (Cell Signaling Technology, Danvers, MA, USA; 5000; 1:200) or Rabbit IgG (Millipore, MA, USA; PP64B; 5 μg) overnight at 4 °C. The mixture was incubated with Protein A + G Agarose/Salmon Sperm DNA for 1 h at 4 °C, washed, and the immunoprecipitated complex was incubated with fresh elution buffer (1% SDS, 0.1 M NaHCO_3_, and 5 M NaCl) at 65 °C for 4 h to reverse cross-linking. DNA purification was performed using Proteinase K and a PCR Clean-Up Kit (Beyotime Biotechnology, Shanghai, China; D0033). The extracted DNA samples were subjected to polymerase chain reaction (PCR) analysis. The ChIP primers are listed in Supplementary Table S1.

### Multiplex Immunohistochemistry (mIHC)

Multiplex immunohistochemistry was performed using an Opal Polaris 7-Color Manual IHC Kit (NEL861001KT) to measure the expression levels of ALYREF, JunD, SLC7A5 and CD8^+^ T cell in the tissue microarray. The dewaxing and rehydration processes were the same as those used for IHC. After rehydration, the slides were fixed in 10% neutral-buffered formalin for 20 min and subjected to antigen retrieval using AR6 buffer in a microwave for 45 s at 100% power and 15 min at 25% power. When the AR6 buffer reached room temperature, the slides were blocked with blocking buffer for 10 min and incubated with primary antibodies for 1 h. Subsequently, the slides were washed three times with TBST and incubated with Opal Polymer HRP Ms+Rb for 10 min. The slides were then incubated with Opal Fluorophore for signal amplification. The procedure of blocking, primary antibody incubation, introduction of Opal Polymer HRP, and signal amplification was repeated until all targets of interest were detected. Finally, 4’,6-diamidino-2-phenylindole (DAPI) solution was used for nuclear staining. Antibody information and the corresponding coupled Tyramide signal amplification (TSA) colors (Opal numbers) are listed below: Pan-cytokeratin (PanCK) (Abcam; ab86734; Opal Polaris 520, 1:300), anti-ALYREF (Cell Signaling Technology, Danvers, MA, USA; 12655; 1:200), CD8 (Abcam, ab237710, 1:400, Opal 690), anti-SLC7A5 (Cell Signaling Technology, Danvers, MA, USA; 32683; 1:200), anti-JunD (Cell Signaling Technology, Danvers, MA, USA; 5000; 1:200).

### Public data and bioinformatics analysis

Differential mRNA expression profiles of m^5^C-related proteins in PDAC and normal pancreatic tissues, based on The Cancer Genome Atlas (TCGA) and Genotype-Tissue Expression (GTEx) databases, were obtained from the Assistant for Clinical Bioinformatics (https://www.aclbi.com/static/index.html#/). The GSE15471 and GSE16515 datasets were downloaded from the Gene Expression Omnibus database (GEO, https://www.ncbi.nlm.nih.gov/geo/). The Kaplan-Meier survival curves were performed with “survival” and “survminer” packages. The Gene Ontology Enrichment Analysis (GO) and Gene Set Enrichment Analysis (GSEA) were conducted by “enrichGO” and “GSEA” packages respectively. All R software packages were from R software version 4.3.1. The potential transcription factors of SLC7A5 are obtained from JASPAR Database (https://jaspar.genereg.net).

### Statistical analysis

Statistical analyses were performed by GraphPad Prism 8.0 (GraphPad, La Jolla, CA, USA) and results were presented as the mean ± SD. The statistical significance of the differences between two groups was evaluated using two-tailed Student’s *t* test, while comparisons among multiple groups were analyzed by one-way analysis of variance (ANOVA). Overall survival was assessed using the Kaplan–Meier method, and the log-rank t-test was used to compare differences. A multivariate Cox proportional hazard regression model was used to explore the independent prognostic factors. The correlation between ALYREF and CD8^+^T cells or between ALYREF, JunD, and SLC7A5 was calculated using Pearson’s correlation coefficient. *P* < 0.05 was defined as statistically significant (*P < 0.05; **P < 0.01; ***P < 0.001; ****P < 0.0001).

### Supplementary information

Supplementary Materials

## Data Availability

All data in this study are available from the corresponding author on reasonable request.
