# Supplementary material for: ALYREF-JunD-SLC7A5 axis promotes pancreatic ductal adenocarcinoma progression through epitranscriptome-metabolism reprogramming and immune evasion
Source: Cell Death Discov. 2024 Feb 24;10:97. doi: 10.1038/s41420-024-01862-2 (PMC10894212; doi:10.1038/s41420-024-01862-2)
Supplement: Supplementary file 1 — Supplementary Materials [file 41420_2024_1862_MOESM1_ESM.pdf]

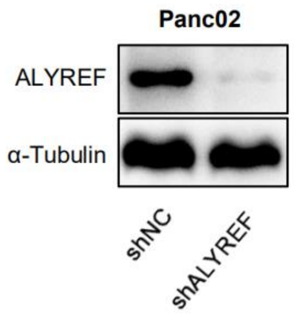

Supplementary Fig. S1. Western blotting proved the knockdown efficiency of ALYREF in Panc02 cells.

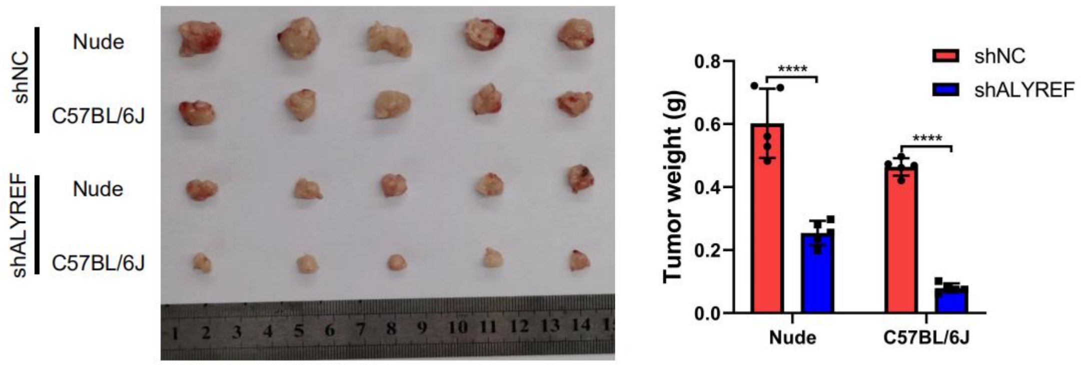

Supplementary Fig. S2. Representative images of tumors and tumor weight in each group.

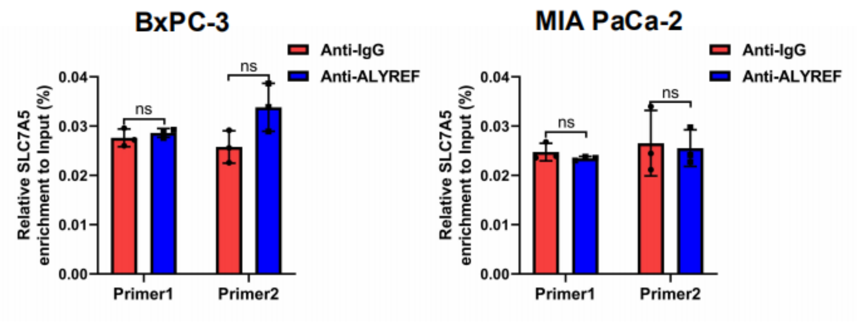

Supplementary Fig. S3. SLC7A5 is not directly targeted by ALYREF. RIP-qPCR results showed that no significant difference was observed between anti-ALYREF and anti-IgG. Thus, ALYREF do not directly regulate the expression of SLC7A5.

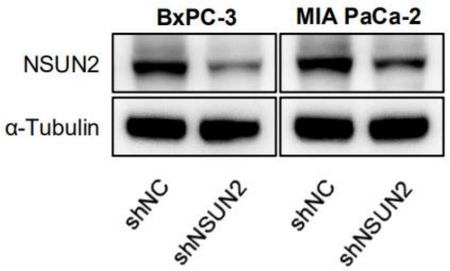

Supplementary Fig. S4. Western blotting proved the knockdown efficiency of NSUN2 in BxPC-3 cells and MIA PaCa-2 cells.

Table S1

**synonymous mutation primers**	
ALYREF-Forward	CATAGAAGATTCTAGGCCACCATGCCCGATTCCGCGCCC
ALYREF-Mut Reverse	TCAGCATCGCTCACGCCAAAATCCAGATTGGACACCAGCAGT
ALYREF-Mut Forward	CGTGAGCGATGCTGACATCCAGGAACTCTTTGCTGAATTTGGAA
ALYREF-Reverse	TCGCGGCCGCGGATCTTAACTGGTGTCCATTCTCGCATTATAGG

**shRNA sequences**	
ALYREF shRNA #1 (human)	GGAGTCTCAGACGCCGATATT
ALYREF shRNA #2 (human)	GAACTCTTTGCTGAATTTGGA
ALYREF shRNA (mouse)	GCTGATATTCAGGAACTCTTT
JunD shRNA #1	GGAGGATTTACACAAGCAGAA
JunD shRNA #2	GACGAGCTCACAGTTCCTCTA
NSUN2 shRNA	GCGATGCCTTAGGATATTATT

**RT-qPCR primers**	
ACTB-Forward	CATGTACGTTGCTATCCAGGC

ACTB-Reverse	CTCCTTAATGTCACGCACGAT
ALYREF-Forward	GGAGTCTCAGACGCCGATATTC
ALYREF-Reverse	GCATCTGCCTTCCGCTCAAAGT
SLC7A5-Forward	GCCACAGAAAGCCTGAGCTTGA
SLC7A5-Reverse	ATGGTGAAGCCGATGCCACACT
ASNS-Forward	CTGTGAAGAACAACCTCAGGATC
ASNS-Reverse	AACAGAGTGGCAGCAACCAAGC
CTH-Forward	CTCACTGTCCACCACGTTCAAG
CTH-Reverse	CAGTGGCTGCTAAACCTGAAGC
TRIB3-Forward	GCTTTGTCTTCGCTGACCGTGA
TRIB3-Reverse	CTGAGTATCTCAGGTCCCACGT
PSAT1-Forward	ACTTCCTGTCCAAGCCAGTGGA
PSAT1-Reverse	CTGCACCTTGTATTCCAGGACC
STC1-Forward	GCAGGAAGAGTGCTACAGCAAG
STC1-Reverse	CATTCCAGCAGGCTTCGGACAA
TM7SF2-Forward	GGTCAATGGCTTCCAGTTGCTC
TM7SF2-Reverse	AACGCCAGCATGAAGCCAAACC
SLC1A4-Forward	TGTGGTTGCAGCTTTCCGTACG
SLC1A4-Reverse	CCAGAGCAAACAGGACCAATCC
CDKN1A-Forward	AGGTGGACCTGGAGACTCTCAG
CDKN1A-Reverse	TCCTCTTGGAGAAGATCAGCCG
IFRD1-Forward	TGCATGGACACTACTGCTGACC
IFRD1-Reverse	GTGCCAAAGATTCACCAGCAGC
JunD-Forward	TCATCATCCAGTCCAACGGG
JunD-Reverse	TTCTGCTTGTGTAAATCCTCCAG

**RIP and MeRIP assay primers**	
JunD RIP-F	AGGACTCGACAAGCTGGAC
JunD RIP-R	GGAAAGGCAGGGTTTGAGG
SLC7A5 RIP-F1	ACTTGTGGGCTTCAGGTGTTT
SLC7A5 RIP-R1	TGGTGCCGACAGCATCTTT

SLC7A5 RIP-F2	GAACTCACCGCTGCCCTCT
SLC7A5 RIP-R2	CGTACTCACCCTTCCCGATCT
JunD MeRIP-F	AGGACTCGACAAGCTGGAC
JunD MeRIP-R	GGAAAGGCAGGGTTTGAGG

**ChIP assay primers**	
CHIP-F1	ATGCCTGCTGTGAGCTTCC
CHIP-R1	AATACTGCCCCGAGATTGACC
CHIP-F2	AGGAGCTTCCCCTCAGGTC
CHIP-R2	GCAGCCTCTCGTCAAGCC
